# I WISH I MAY, I WISH I MIGHT FEEL THE AGE I WISH TONIGHT

**DOI:** 10.1093/geroni/igac059.642

**Published:** 2022-12-20

**Authors:** Jennifer Bellingtier, Fiona Rupprecht, Shevaun Neupert, Frieder Lang

**Affiliations:** Friedrich Schiller University Jena, Jena, Thuringen, Germany; University of Vienna, Vienna, Wien, Austria; North Carolina State University, Raleigh, North Carolina, United States; Friedrich-Alexander-Universität Erlangen-Nürnberg, Nürnberg, Bayern, Germany

## Abstract

Subjective age has traditionally been considered by comparing felt age to chronological age, with those who feel younger reporting more adaptive developmental outcomes. Here we consider a new approach: subjective age discordance, which compares felt ages to the ideal ages of participants. Across eight study days, 116 older and 107 younger adults reported their daily felt and ideal ages. On the majority of days, both older and younger adults idealized ages younger than they felt. The opposite pattern, idealized ages older than felt ages, was rare and primarily seen in younger adults. Days when felt ages were less discordant from ideal ages were characterized by higher levels of positive affect than days with greater subjective age discordance. These findings suggest that positive developmental outcomes can occur not only from feeling younger, but through a greater alignment of ideal and felt ages.

